# Supplementary material for: Significant Enrichment of Potential Pathogenic Fungi in Soil Mediated by Flavonoids, Phenolic Acids, and Organic Acids
Source: J Fungi (Basel). 2025 Feb 17;11(2):154. doi: 10.3390/jof11020154 (PMC11856650; doi:10.3390/jof11020154)

Supplementary

Table S1. The composition of various root secretions

Types of secretion	Component	mg/d
Flavonoids	Apigenin	1.689
	Hesperidin	1.783
	Coumarin	1.522
	Naringin	1.702
Phenolic acids	P-hydroxybenzoic acid	2.467
	Salicylic acid	2.466
	Ferulic acid	2.427
Organic acids	Citric acid	4.002
	Tartaric acid	4.69
	Succinic acid	3.69

Table S2. Relative abundance of rhizosphere soil fungi dominant microbial in different treatments (Genus level)

Genus（%）	*Fusarium*	*Saitozyma*	*Mortierella*	*Penicillium*	*Trichoderma*
CK	11±7.08c	20.96±14.14a	10.34±6.07a	1.86±2.34a	1.38±1.01b
FLA	66.45±21.11a	11.4±7.31ab	1±2.06b	0.69±0.85a	0.09±0.11c
OA	61.97±35.88a	1.71±1.79b	0.24±0.32b	0.61±0.61a	0.38±0.24bc
PA	47.58±33.42ab	0.05±0.02b	0.02±0.01b	2.49±2.48a	0.26±0.54bc

NOTE: CK: control (sterile water), FLA: Flavonoids; OA: organic acids, PA: phenolic acids. Different letters indicate significant differences between treatments under the same soil microbes ($p < 0.05$).

Table S3. Relationship and Explained Variance of Soil Factors for Major Fungal Groups

Treatment	R^2	*p* value
S-PPO	0.016535	0.82059
S-CL	0.361597	0.011494
S-UE	0.712303	0.0005
S-CAT	0.02897	0.725137
S-POD	0.285603	0.029485
S-DHA	0.192842	0.109445
S-ACP	0.296982	0.023488
S-ALP	0.3522	0.004998
SOM	0.194765	0.095952
PH	0.158543	0.170915
AK	0.234202	0.058471
AP	0.193983	0.097451
AHN	0.515066	0.0005

Note: S-PPO (nmol/h/g): soil polyphenol oxidase; S-CL (μg/d/g): soil-cellulase; S-UE (μg/d/g): soil urease; S-CAT (μmol/h/g): soil catalase; S-POD (nmol/h/g): soil peroxidase; S-DHA (μg/d/g): soil dehydrogenase; S-ACP (nmol/h/g): soil acid phosphatase; S-ALP (nmol/h/g): soil alkaline phosphatase; SOM (g/kg): soil organic carbon; AK (mg/kg): soil-available potassium; AP (mg/kg): soil-available phosphorus; AHN (mg/kg): alkali-hydrolyzed nitrogen. Different letters indicate significant differences between treatments ($p < 0.05$). n=3.

Figure S1. Microecological characteristics of aseptic water treatment and 20% sterile methanol solution

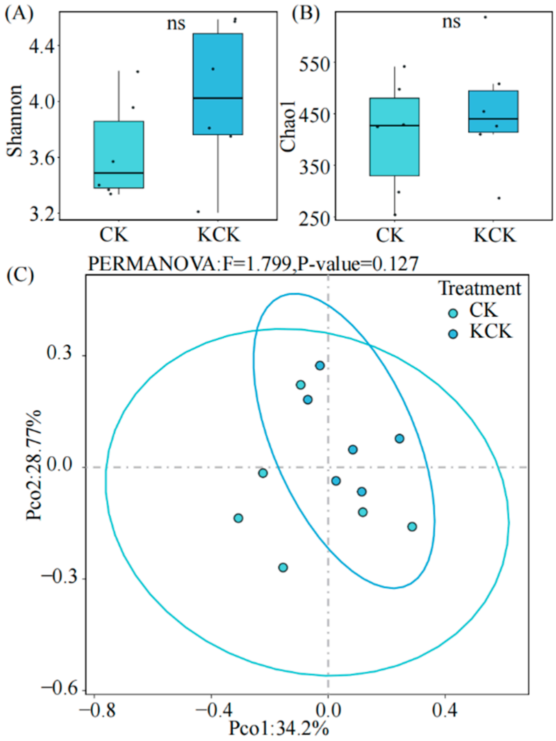

Supplement: Supplementary file 1 [file jof-11-00154-s001.zip › jof-3457871-supplementary.pdf]
